# A β-Glucan-Based Dietary Fiber Reduces Mast Cell-Induced Hyperpermeability in Ileum From Patients With Crohn’s Disease and Control Subjects

**DOI:** 10.1093/ibd/izx002

**Published:** 2017-12-19

**Authors:** John-Peter Ganda Mall, Maite Casado-Bedmar, Martin E Winberg, Robert J Brummer, Ida Schoultz, Åsa V Keita

**Affiliations:** 1 School of Medical Sciences, Nutrition-Gut-Brain Interactions Research Centre, Örebro University, Örebro, Sweden; 2 Department of Clinical and Experimental Medicine, Linköping University, Linköping, Sweden

**Keywords:** β-glucan, Crohn’s disease, intestinal permeability

## Abstract

**Background:**

Administration of β-glucan has shown immune-enhancing effects. Our aim was to investigate whether β-glucan could attenuate mast cell (MC)-induced hyperpermeability in follicle-associated epithelium (FAE) and villus epithelium (VE) of patients with Crohn’s disease (CD) and in noninflammatory bowel disease (IBD)-controls. Further, we studied mechanisms of β-glucan uptake and effects on MCs in vitro.

**Methods:**

Segments of FAE and VE from 8 CD patients and 9 controls were mounted in Ussing chambers. Effects of the MC-degranulator compound 48/80 (C48/80) and yeast-derived β-1,3/1,6 glucan on hyperpermeability were investigated. Translocation of β-glucan and colocalization with immune cells were studied by immunofluorescence. Caco-2-cl1- and FAE-cultures were used to investigate β-glucan-uptake using endocytosis inhibitors and HMC-1.1 to study effects on MCs.

**Results:**

β-glucan significantly attenuated MC-induced paracellular hyperpermeability in CD and controls. Transcellular hyperpermeability was only significantly attenuated in VE. Baseline paracellular permeability was higher in FAE than VE in both groups, *P*<0.05, and exhibited a more pronounced effect by C48/80 and β-glucan *P*<0.05. No difference was observed between CD and controls. In vitro studies showed increased passage, *P*<0.05, of β-glucan through FAE-culture compared to Caco-2-cl1. Passage was mildly attenuated by the inhibitor methyl-β-cyclodextrin. HMC-1.1 experiments showed a trend to decreasing MC-degranulation and levels of TNF-α but not IL-6 by β-glucan. Immunofluorescence revealed more β-glucan-uptake and higher percentage of macrophages and dendritic cells close to β-glucan in VE of CD compared to controls.

**Conclusions:**

We demonstrated beneficial effects of β-glucan on intestinal barrier function and increased β-glucan-passage through FAE model. Our results provide important and novel knowledge on possible applications of β-glucan in health disorders and diseases characterized by intestinal barrier dysfunction.

The importance of a normal barrier function is widely recognized as several gastrointestinal complaints and diseases are correlated with a decrease in barrier function and increased gut permeability.^1–4^ Crohn’s disease (CD) is an inflammatory bowel disease (IBD) characterized by a chronic transmural inflammation, anywhere from the oral cavity to the anus, which might originate from the stimulation of an exaggerated mucosal immune system by either a normal or dysbiotic commensal bacterial gut flora.^4^ It is known that patients with CD have an increased gut permeability,^5^ which might lead to increased passage of luminal bacteria, endotoxins, and antigens that in turn have further negative effects on the barrier function.

The first observable signs of CD are microscopic erosions in the follicle-associated epithelium (FAE), overlying the Peyer’s patches in the human ileum.^6^ The cell composition of FAE differs from the surrounding villus epithelium (VE).^7,8^ FAE has a biochemical face to the lumen that facilitates uptake of antigen and various microorganisms,^9^ and the uptake capacity in FAE is further promoted by the presence of the highly specialized microfold or membranous (M) cells.^10^ The M cells are adapted to translocate luminal contents into juxtaposition with cells of the immune system beneath the FAE, such as dendritic cells (DCs), macrophages, and mast cells (MCs). We previously showed that human FAE and VE differ in transcellular permeability for the antigen horseradish peroxidase (HRP) and both live and heat-killed fluorescent *Escherichia (E.) coli*.^11^ FAE displayed higher HRP and *E. coli* translocation compared to VE. Further, we demonstrated that patients with CD have a greater affinity for the translocation of antigens and nonpathogenic *E. coli* in both FAE and VE compared to non-IBD controls.^12^

MCs have shown to be important in the regulation of intestinal barrier function and IBD pathophysiology.^13–16^ Upon activation, MCs release cytokines and mediators, particularly TNF-α, IL-6, and proteases that alter the expression of tight junction proteins such as ZO-1 and claudin-2^17–19^ and facilitate cytoskeletal contraction leading to increased paracellular permeability.^20^ MC degranulation also can lead to increased transcellular passage as shown via enhanced uptake of HRP in colonocyte endosomes.^21^ As reviewed by Bischoff et al,^22^ an increased number of MCs and their activity have been reported in patients with CD. Moreover, Bischoff et al showed increased numbers of eosinophils in CD colon, and it was previously demonstrated^23^ that eosinophils release corticotrophin-releasing hormone upon activation, which in turn degranulates MCs leading to increased intestinal permeability. Patients with CD have shown increased number of MCs in mucosal sections from both inflamed and uninflamed ileum compared to non-IBD controls.^24^

Although the trigger for MC degranulation during CD is unknown, the link between intestinal permeability and MC degranulation has been widely demonstrated,^14,21,25^ and could be one of the main reasons behind the increased gut permeability seen in patients with CD.

Pro- and prebiotic supplementation is able to modulate the gut microbiota and its metabolic products and has emerged as an attractive, cost-effective therapeutic for patients and clinicians in the treatment of gut-related disorders such as IBD.^26^ However, little is known about the direct effects these substances exert on the intestinal epithelium and barrier function. Beta (β)-glucan, a prebiotic fiber from Baker’s yeast found in the cell wall of the yeast strain *Saccharomyces cerevisiae*, has in several studies shown immune-enhancing effects in both cancer therapy and resistance towards cold-induced infection.^27–31^ Furthermore, transportation of β-glucan to murine lymphoid tissues seemed to involve gastrointestinal macrophages.^28^ Although these studies provide evidence for immune-enhancing capabilities of β-glucan administration, no study has to our knowledge demonstrated any beneficial effect on the human intestinal barrier function ex vivo.

Given the increased use of prebiotics world-wide, particularly in the treatment of gut-related disorders, it is essential to conduct advanced studies to understand the underlying mechanisms of how these substances affect the intestinal barrier function.

We hypothesized that β-glucan has a beneficial effect on intestinal barrier function in patients with CD. Using Ussing chambers we aimed to evaluate whether a specific yeast- derived β-glucan could attenuate MC-induced intestinal hyperpermeability in patients with CD ileitis and in non-IBD controls. Further, we performed in vitro experiments to study mechanisms of (1) β-glucan uptake using the endocytosis inhibitors methyl-β-cyclodextrin (MβCD) and chlorpromazine (CPZ), and (2) the effects of β-glucan on MC degranulation and cytokine secretion.

## MATERIALS AND METHODS

### Ethical consideration

The study was approved by the Committee of Human Ethics, Linköping, and all subjects had given their informed written consent. The sample size was determined based on previous Ussing chamber studies.^11,12^

### Patients

Specimens from the terminal or neoterminal ileum were obtained during surgery between 2015–2017 at Linköping University Hospital from 8 patients with ileal CD and 9 patients with colonic cancer, as non-IBD controls. The CD patients, 4 men and 4 women, had a median age of 51 years (range 20–59) and disease duration of 13 years (range 5–37). According to the Montreal classification, all patients were in an active phase of the disease; however, tissue obtained for Ussing experiments were dissected from mild to noninflamed ileum. The non-IBD control group included microscopically normal ileal specimens from 5 men and 4 women, median age 72 years (range 43–82). None of the cancer patients had signs of generalized disease and none had received preoperative chemo- or radiotherapy.

### Ussing Chamber Experiments in Humans

Surgical specimens from 7 CD and 8 controls were put in cold oxygenated Krebs buffer immediately after division of the ileocolic artery, transported to the laboratory, and stripped of external muscle layers. Segments of VE and FAE were identified in a dissection microscope and mounted in Ussing chambers as previously described.^11^ A concentration of 0.5 mg/ml soluble β-1,3/1,6-glucan from Baker’s yeast (Biothera, Eagan, MN, USA) was added to the mucosal sides of chambers 1–2. After 20 minutes, 10 ng/ml compound 48/80 (C48/80) (Sigma Chemical Co, MO, USA) was added to the serosal sides of chambers 1–4. The chemical C48/80 is known to act as a stressor on the epithelium by degranulating MCs and has been used in Ussing chambers to induce MC degranulation and increased intestinal permeability.^25,32–36^ Chambers 5–6 served as controls with only buffer added. The paracellular marker FITC-dextran 4000 (Sigma) and the 45 kD transcellular marker HRP (Type VI; Sigma) were added to the mucosal sides at 2.5 nM and 10^−5^ M, respectively. Serosal samples were collected at 0 and 90 minutes. The short-circuit current (Isc), transepithelial resistance (TER), and transepithelial potential difference (PD) were monitored throughout the experiments to ensure good tissue viability. Three FAE and 6 VE segments did not reach cut-off values set for viability and were excluded from further analyses.

Before experiments, concentrations of C48/80 and β-1,3/1,6-glucan were optimized. Effects of C48/80 were evaluated at 5, 10, and 50 ng/ml and the highest peak in permeability was achieved with 10 ng/ml. Concentrations of 0.5, 5, and 50 mg/ml of soluble β-1,3/1,6-glucan were then evaluated against 10 ng/ml C48/80 and results showed that 0.5 mg/ml was the optimal concentration.

### Microscopic Studies of Yeast-derived β-glucan Uptake

To study the uptake of β-glucan and its interaction with immune cells, FAE and VE from 5 non-IBD controls and 5 CD patients were mounted in Ussing chambers and 0.5 mg/ml of Alexa Fluor 594-conjugated soluble β-1.3/1,6-glucan (Biothera, Eagan, MN, USA) was added to the mucosal sides. After 20 minutes, chambers were washed with PBS to remove nonadherent β-glucan. Tissues were fixed in the chambers with 4% formaldehyde overnight, carefully removed, embedded in OCT mounting medium (Sakura-Finetek, The Netherlands), and cryosectioned at 10 µm.

Serial sections of FAE and VE were stained separately with monoclonal antibodies to MCs, macrophages, or DCs. Slides were incubated with background sniper (Histolab, Sweden) for 7 minutes, washed in PBS, and incubated overnight at 4°C with monocolonal antibodies mouse-anti-human MC tryptase 1:200 (Santa Cruz Biotechnology, Heidelberg, Germany), macrophage marker CD68 1:100 (Abcam, Cambridge, UK), or DC-SIGN 1:100 (Invitrogen). Slides were rinsed in PBS and incubated with Alexa Fluor 488-conjugated rabbit-anti-mouse (1:400 Life Technologies, OR, USA) for 1 hour at room temperature (RT). Slides were rinsed and mounted with Prolong® Gold/DAPI (Invitrogen, Sweden). Negative controls with secondary/primary antibodies only were included in all experiments with no unspecific binding confirmed.

Slides were blindly evaluated in Nikon E800 fluorescence microscope connected to software NIS-elements (Nikon Instruments Inc. Tokyo, Japan). First, the amount of uptaken β-glucan was manually quantified in 8–10 areas per section (each 0.069 mm^2^) at 40x objective. Second, the total numbers of macrophages, MCs, and DCs were quantified in the same way. In order to achieve a picture of how many of the immune cells colocalized with β-glucan, the number of macrophages, MCs, and DCs found in close proximity (defined as direct colocalization or <1 µm) to β-glucan were quantified, and then the percentage of colocalizing cells was calculated.

### Analysis of FITC-dextran and HRP

FITC-dextran passage was determined at 488 nm in a VICTOR *X3* multileader plate reader. HRP passage was measured with QuantaBlu™ Fluorgenic Peroxidase Subtrate Kit (Pierce, Rockford, USA). A 96-well plate was coated with carbonatebuffer (pH 9.6)+5 mg/ml anti-HRP mouse IgG monoclonal antibody (GenWay, USA). After 4°C overnight incubation, the plate was washed with PBS-Tween and incubated with 5% BSA blocking buffer, 500 rpm, for 1 hour. After washing, standards and samples were loaded and incubated for 1 hour at 300 rpm followed by washing. Wells were added glucose-Krebs+0.2 mg/ml BSA and working-solution. After 30 minutes of dark incubation at 300 rpm, stop-solution was added, plate was incubated in dark for 10 minutes, 300 rpm, and measured at λ_ex_ = 315nm, λ_em_ = 470nm using EnSpire® Multimode Plate Reader (Perkin Elmer, MA, USA). Results from the FITC-dextran and HRP-analyses were expressed as Δ90-0 minutes, and samples were measured in duplicates and measured against a standard curve.

### In vitro Studies of Yeast-derived β-glucan Using a Coculture Model of Human FAE

#### Coculture Model Setup

To study the translocation of β-glucan through human FAE, an in vitro coculture model was used. Coculture of Peyer’s patch lymphocytes and intestinal epithelial cells can trigger epithelial cell conversion to an M cell-like phenotype,^37^ and we previously established a modified version of this coculture.^14^ Briefly, intestinal epithelial Caco-2-cl1 cells (originally obtained from Dr. Maria Rescigno, Italy) were grown for 14–17 days on Matrigel-coated (Becton Dickinson, USA) 3.0 μm polycarbonate filters (Costar, Baedvenhorp, NL) until reaching confluence. The model FAE was obtained by adding 5x10^5^ Raji B cells (ATCC, ML, USA) suspended in DMEM-supplemented media to the basolateral chamber of confluent Caco-2-cl1 monolayers. Corresponding monocultures of Caco-2-cl1 cells on matched filter supports served as controls. The coculture was maintained for 4–6 days until M cells were generated. To confirm transformation to M cells, TER was measured daily, and to be considered transformed, TER should be at least 10% lower in the coculture model compared to the monoculture.^14^

#### Studies of Yeast-derived β-glucan Translocation

Translocation experiments were performed in Hanks’ Balanced Salt Solution (HBSS) supplemented with 10% fetal bovine serum. Before and after experiments, TER was measured to check cell monolayer integrity. To study uptake mechanisms of β-glucan, two endocytosis inhibitors were used; MβCD, which chelates cholesterol from the plasma membrane,^38^ has previously been shown to decrease the translocation of β-glucan expressing fungi by inhibiting lipid raft formation.^39^ The other inhibitor, CPZ, inhibits endocytosis by a reversible translocation of clathrin and its adapter proteins, thus, hindering clathrin-mediated vesicle formation.^40^

Upon M cell-transformation, monolayers were washed and preincubated with HBSS with or without the addition of 3 mM MβCD (Sigma-Aldrich) or 30 µM CPZ (Fluka) at 37°C for 20 minutes. Both cultures were then apically exposed in duplicates to 0.5 mg/ml of Alexa Fluor 594-conjugated β-glucan for 20 minutes and 1 hour. Basolateral media were collected in triplicates and β-glucan fluorescence was measured in Modulus Microplate Photometer (λ_ex_ = 525nm, λ_em_ = 580–640 nm). The fluorescence intensity data generated from these measurements were used for subsequent statistical analysis.

For microscopy, filters were washed with PBS and fixed with 4% formaldehyde for 30 minutes, RT. After washing, cells were incubated with Phalloidin 488 (Santa Cruz), 1:1000 in PBS+1% BSA for 30 minutes, RT. Filters were washed, cut, and mounted with ProLong Gold, and translocation of β-glucan was studied using Zeiss LSM 700 Inverted Confocal Microscope.

### In vitro Studies of MCs

To study the effects of C48/80 on degranulation and cytokine secretion by MCs, and the impact of β-glucan, the human mast cell line HMC-1.1 (Merck Chemicals and Life Science AB, Sweden) was used. Cells were cultured in Isove Medium (EMD, Millipore) supplemented with 1.2 mM α-thioglycerol (Sigma), 10% fetal bovine serum, and 1% penicillin/streptomycin at 37°C in a 5% CO2 incubator. In vitro experiments for MC degranulation were set as follows: 10^6^ HMC-1.1 cells/500 µl in DPBS were incubated in duplicates with or without 0.5 mg/ml β-glucan for 20 minutes followed by incubation with C48/80 at an optimal concentration of 20 µg/ml. Wells with HMC-1.1 cells added DPBS only served as negative controls. After 24 hours, samples were centrifuged at 300*g* for 4 minutes and supernatants were collected for MC mediator release and cytokine measurements.

Before experiments, concentrations of C48/80 and β-glucan were optimized. Degranulation effects of C48/80 were evaluated at concentrations of 10 ng/ml, 100 ng/ml, 10 µg/ml, and 20 µg/ml during 12 and 24 hours, and the optimal degranulation, measured by the β-Hexosaminidase assay described below, was achieved with 20 µg/ml C48/80 for 24 hours. Concentrations of 0.5 and 1 mg/ml of soluble β-glucan were evaluated against 20 µg/ml C48/80, and results showed that 0.5 mg/ml was the optimal concentration. To illustrate the interaction between MCs and β-glucan, experiments above were performed with Alexa Fluor 594-conjugated β-glucan. After centrifugation, pellets were washed with PBS, fixed in 4% formaldehyde, washed again, and cytocentrifuged at 300*g* for 5 minutes. Smeared cells on slides were then stained for MC tryptase according to the same protocol as described for tissues.

#### β-hexosaminidase Assay

Sample aliquots of 50 µl were incubated in duplicates with 200 µl of 1mM p-nitrophenyl N-acetyl-β-D-glucosamine in 0.05 M citrate buffer (pH 4.5) for 1 hour at 37°C. Cells lysed with 1 % Triton X-100 served as positive control. The reaction was quenched by adding 500 µl of 0.05 M sodium carbonate buffer (pH 10.0). Optical density was read at 405 nm with VERSAmax Tunable Microplate Reader (Molecular Devices, CA, USA) using Softmax pro 5 (Molecular Devices).

#### Cytokine Measurement by ELISA

To examine the effects of β-glucan on cytokine secretion by MCs, the supernatants of HMC-1.1 stimulated cells, with or without C48/80 and/or β-glucan, were analyzed in duplicates for TNF-α (Human ultrasensitive TNF-α, Invitrogen, Fisher Scientific, Sweden) and IL-6 (Human IL-6, Thermo Scientific, Fisher Scientific) according to manufacturer’s instructions. Optical density was read at 450 nm with VERSAmax Tunable Microplate Reader with a standard curve based on the standard points in duplicates, from which the concentrations of the samples were calculated. Cells lysed with 1 % Triton X-100 served as positive control.

### Statistics

Nonparametric data are presented as scatter plots with median, or in the text as median (25^th^–75^th^ percentile), and comparisons between two groups were done using Mann-Whitney *U* test.

In vitro data, confirmed as normally distributed by log_2_-transforming the data and plotting as histograms, are presented as mean ± SD. A two-way ANOVA was used to analyze the results of the in vitro studies. Log-values are modeled by two factors, experiment and treatment/cell type, meaning that the method compensates for the influence of the experiment and then tests for treatments/cell type effect. When the difference between experiments was not significant, the analyses of treatment/cell type effect were run as Students *t* test. Differences of *P* < 0.05 were considered significant.

## RESULTS

### Ussing Chamber Experiments

#### C48/80-induced Paracellular Hyperpermeability Is Attenuated by Yeast-derived β-glucan

**FIGURE 1.**
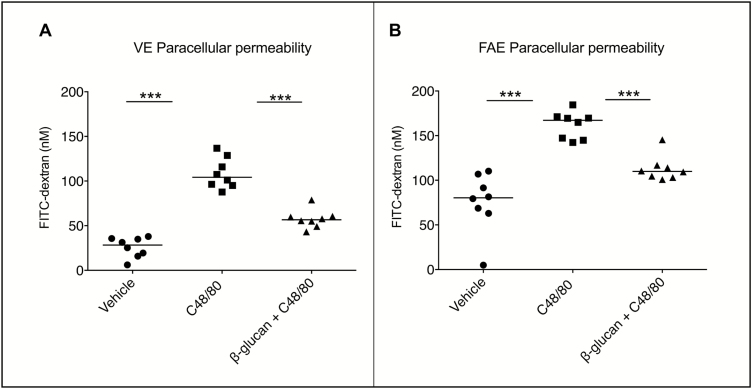
Effects of yeast-derived β-glucan on compound 48/80 (C48/80)-induced paracellular hyperpermeability in VE and FAE of 8 control subjects mounted in Ussing chambers. (A-B) Control subjects displayed an increased FITC-dextran passage in VE and FAE after stimulation with the mast cell degranulator C48/80 compared to unstimulated tissues (vehicles). Costimulation with β-glucan attenuated C48/80 effects to levels close to vehicles. A similar pattern was seen for CD patients (Suppl Fig. 1). Data (∆90—0 min) are presented as a line intersecting the median and each dot representing one patient, ***P < 0.001

In the non-IBD controls, both VE and FAE stimulated with C48/80 resulted in 2–3 times significantly higher paracellular permeability, compared to unstimulated tissues (vehicle) after 90 minutes (Fig. 1A–B). The C48/80-induced increase in paracellular permeability was significantly attenuated in VE and FAE added C48/80+β-glucan. No difference in response towards C48/80 and β-glucan could be observed in the CD patients compared to the controls, as similar results were evident in both VE (vehicle: 38.0 nM, C48/80: 110.7, C48/80+β-glucan: 50.2) and FAE (vehicle: 57.8, C48/80: 149.9, C48/80+β-glucan: 67.7) of the CD patients. There was a higher baseline paracellular permeability in FAE compared to VE in both CD patients and controls (*P* < 0.05) and a more pronounced effect of C48/80 and β-glucan (*P* < 0.05). The effects of C48/80 and β-glucan on FITC-dextran permeability for each patient/control separately can be viewed in Supplementary Figure 1. There were no significant differences in TER or Isc between any of the groups (Supplementary Table 1).

#### C48/80-Induced Increase in Transcellular Passage Is Attenuated by Yeast-derived β-glucan

**FIGURE 2.**
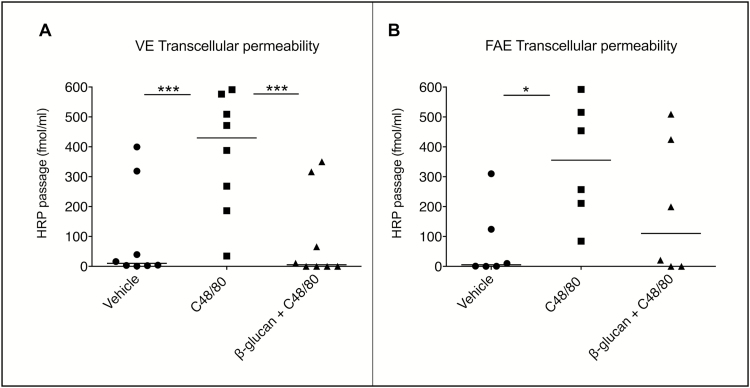
Effects of yeast-derived β-glucan on compound 48/80 (C48/80)-induced transcellular hyperpermeability in VE and FAE of 6 control subjects mounted in Ussing chambers. (A-B) Control subjects displayed an increased HRP passage in VE after stimulation with the mast cell degranulator C48/80 compared to unstimulated VE (vehicle). Costimulation with β-glucan attenuated C48/80 effects to levels close to vehicle. In FAE, there was a significant increase in HRP passage after C48/80 stimulation, however, costimulation with β-glucan showed no effect. A similar pattern was seen for CD patients, although lacking significance in FAE (Suppl Fig. 2). Data (∆90—0 min) are presented as a line intersecting the median and each dot representing one patient. *P < 0.05, ***P < 0.001

In VE of non-IBD controls, C48/80 significantly increased the transcellular passage, and this increase was significantly diminished by simultaneous addition of β-glucan (Fig. 2A). The same pattern was seen in CD patients (vehicle: 3.2 fmol/ml, C48/80: 191.2, C48/80+β-glucan: 6.0). In FAE, however, transcellular passage was significantly increased by C48/80 only in control subjects (Fig. 2B), even though there was a trend towards significance in CD patients (vehicle: 82.5, C48/80: 121.7, C48/80+β-glucan: 110.8), *P* = 0.07. The diminishing effect of β-glucan was nonsignificant in both groups. The effects of C48/80 and β-glucan on transcellular passage for each patient/control separately can be viewed in Supplementary Figure 2. Even if there were no significant effects of C48/80 in the FAE of CD patients, and no effect of β-glucan in neither of the groups, 4/7 CD patients showed a clearly increased HRP passage by C48/80-stimulation (Supplementary Fig. 2B). Further, an attenuation of this increase by β-glucan was seen in 4/7 CD patients and 4/6 controls (Supplementary Figs. 2B, D). Two controls were excluded from HRP-analysis in FAE due to technical problems; therefore, the number of controls for HRP results was 6 instead of 8 in FAE.

### Immunofluorescence

**FIGURE 3.**
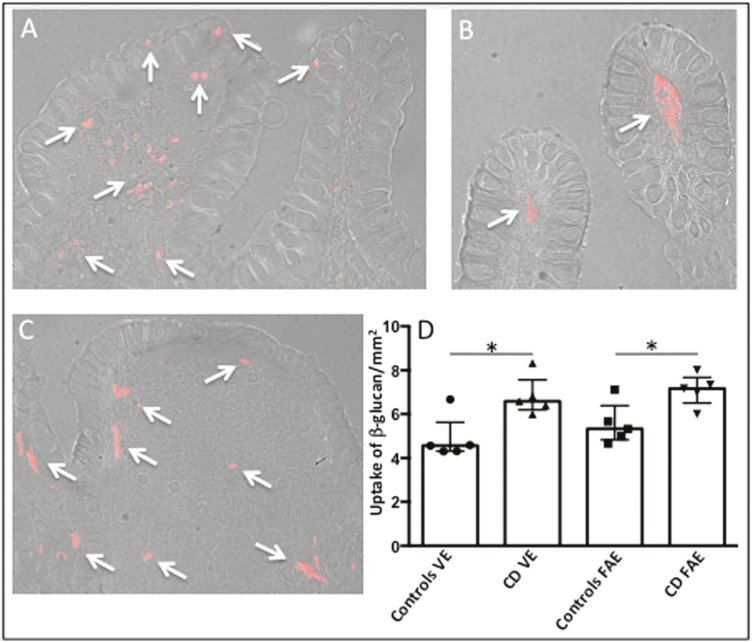
Uptake of Alexa Fluor 594-conjugated yeast-derived β-glucan into the VE and FAE of patients with CD and control subjects, after 20 minutes in Ussing chambers. (A) Uptake of β-glucan (red, arows) in the VE of a control subject. (B) Uptake of β-glucan (red, arrows) in the VE of a CD patient (C) Uptake of β-glucan (red, arrows) in the FAE of a CD patient. Photographs show β-glucan close to the epithelial cell lining as well as further down in the underlying lamina propria/subepithelial dome and follicle. Photographs are in 200X magnification. (D) Quantification of β-glucan uptake. Values are presented as median (25^th^-75^th^ percentile) and comparisons between groups were done with Mann- Whitney *U* test, *P < 0.05

Fluorescence microscopy revealed β-glucan uptake in both VE and FAE after 20 minutes in Ussing chambers (Figs. 3A–C). β-glucan was found not only directly under the epithelium, but also further down in the lamina propria and Peyer’s patches. Quantification revealed significantly more uptaken β-glucan in both VE and FAE of CD compared to non-IBD controls, *P* < 0.05, while there was no statistical difference between VE and FAE within the patient groups (Fig. 3D).

**FIGURE 4.**
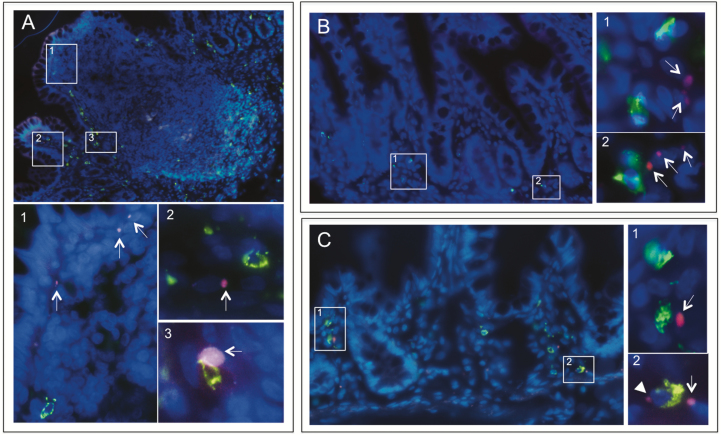
Fluorescence microscopy of Alexa Fluor 594-conjugated yeast-derived β-glucan translocating through the VE and FAE from patients with CD and control subjects. After 20 minutes in Ussing chambers, tissues were stained for MC tryptase or CD68. (A) Staining of MCs (green) in Peyer’s patches from a CD patient showing β-glucan (red, arrows) close to the FAE (1), within the adjacent villi to the FAE (2) and occasionally colocalizing with MCs . (B) Staining of MCs (green) in VE of a CD patient showing β-glucan (arrows) in close proximity to MCs (1–2). (C) Staining of CD68 in VE from a control patient showing β-glucan (red, arrows) in close proximity to macrophages (green) (1- 2), or in a direct colocalization with macrophages (2, arrowhead). Overview photographs are in 400X magnification and zoomed pictures in 1000X

Staining for MC tryptase and CD68 showed β-glucan in close proximity to both macrophages and MCs (Figs. 4A–C). A colocalization of β-glucan with macrophages was generally seen while a direct colocalization with MCs was more rare (Figs. 4A3, C2).

**FIGURE 5.**
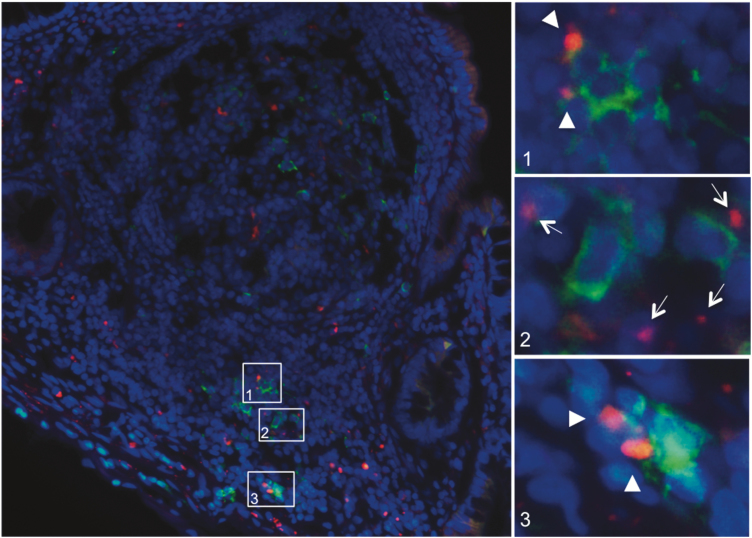
Fluorescence microscopy of Alexa Fluor 594-conjugated yeast-derived β-glucan translocating through the FAE and underlying Peyer’s patches from a patient with CD. After 20 minutes in Ussing chambers, tissue was stained for the DC marker ^DC-SIGN^. Microscopy revealed DCs^DC-SIGN+^ (green) in the subepithelial dome and further down in the Peyer’s patches at follicle margins in close proximity to β-glucan (1–3, red, arrows). Windows 1 and 3 illustrate a direct colocalization between β-glucan and DCs^DC-SIGN+^ (arrowheads). Overview photographs are in 400X magnification and zoomed pictures in 1000X

Staining for DC-SIGN showed β-glucan in close proximity to DCs^DC-SIGN+^ and a direct colocalization could frequently be observed (Fig. 5). Quantification of immune cells showed significantly higher numbers of macrophages, MCs, and DCs^DC-SIGN+^ in VE of CD patients compared to controls, whereas in FAE there were only significantly increased amounts of macrophages (Table 1). Further, there were significantly more MCs in VE compared to FAE in both CD and controls, although there were no significant differences in the amount of macrophages and DCs.^DC-SIGN+.^

**Table 1:** Quantification of Immune Cells and Proximity to Uptaken β-glucan

Number of Positive Cells/mm^2^	Controls VE	CD VE	Controls FAE	CD FAE
Macrophages (CD68)	**77** (72–110)	**122** (88–138)*	**48** (46–59)	**76** (63–92)*
MCs (MC tryptase)	**78** (57–103)§	**105** (101–156)*§	**52** (48–60)	**60** (46–70)
DCs^DC-SIGN+^	**44** (33–47)	**58** (51–61)*	**36** (33–48)	**52** (42–62)
Proximity to β-glucan (% of cells)	Controls VE	CD VE	Controls FAE	CD FAE
Macrophages+β-glucan	**10.1** (8.8–10.9)	**12.2** (10.9–16.2)^#^	**23.1** (14.4–26.1)	**19.1** (15.9–24.5)^€^
MCs+β-glucan	**6.6** (5.5–8.2)	**7.8** (6.7–11.0)	**8.3** (7.1–18.3)	**12.5** (6.0–17.4)^€^
DCs^DC-SIGN+^+β-glucan	**29.1** (24.7–36.6)	**39.3** (33.6–46.7)^#^	**33.3** (29.3–42.7)	**38.5** (34.9–53.1)

Segments of VE and FAE from 5 patients with CD and 5 non-IBD controls were mounted in Ussing chambers and added Alexa Fluor 594-conjugated β-glucan to the mucosal sides for 20 minutes. After embedding and sectioning, the sections were stained for CD68, MC tryptase, and (DCs)^DC-SIGN+^. The number of positive cells were quantified and presented as number of positive cells/mm^2^. Further, the percentage of the cells found in close proximity to uptaken β-glucan was calculated. Values are presented as median (25^th^-75^th^ percentiles), comparisons were made with Mann-Whitney ***U*** test, and *=*P* < 0.05 compared to VE of controls and FAE of controls, respectively; §=*P* < 0.05 compared to FAE of controls and CD, respectively; #=*P* < 0.05 compared to VE of controls; €=*P* < 0.05 compared to VE of controls and CD, respectively.

Quantification of uptaken β-glucan colocalizing with immune cells revealed significantly higher percentages of both DCs^DC-SIGN+^ and macrophages in close proximity to β-glucan in the VE of CD compared to controls, whereas there were no differences in FAE (Table 1). Moreover, the percentage of macrophages in close proximity to β-glucan was significantly higher in the FAE of both CD and controls compared to VE. There was no significant difference in the percentage of MCs in close proximity to β-glucan, between CD and controls, neither in VE nor in FAE, and no difference between the epithelial types within the groups (Table 1).

### In vitro Experiments

#### Higher Transport of Yeast-derived β-glucan Through the FAE Coculture Model

**FIGURE 6.**
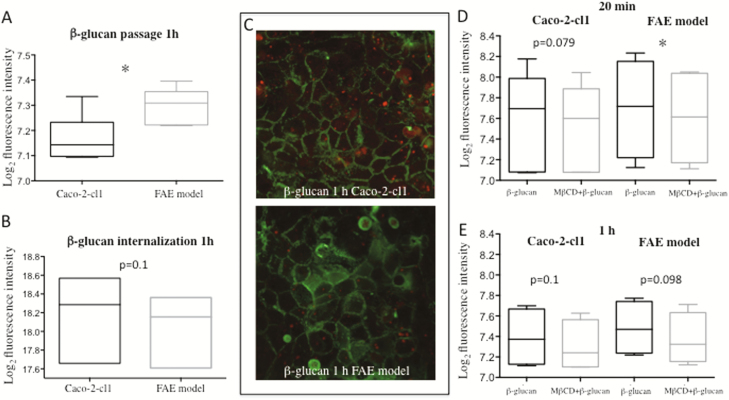
Passage and translocation of Alexa Fluor 594-conjugated yeast-derived β-glucan through monolayers of Caco-2-cl1 and FAE coculture model after 1 hour incubation. (A) β-glucan passage through the in vitro models (7 independent experiments with duplicates). (B) Intracellular β-glucan quantification by confocal microscopy of in vitro models (3 independent experiments with duplicates, 7 Z-stack areas each). (C) Representative photograph of cells stained with phalloidin (green) and translocating β-glucan (red) in the Caco2-cl1 and FAE coculture model, 63X magnification. (D-E) β-glucan passage through Caco2-cl1 and FAE model after 20 minutes incubation (D) and 1 hour incubaction (E) with and without 20 minutes preincubation with the lipid raft inhibitor MβCD (4 independent experiments with duplicates). *P < 0.05. All data (A-E) were analyzed on log_2_-scale with FAE model compared to Caco2-cl1 (A-B) and the MβCD-treated groups (D-E) compared to the untreated groups of the Caco-2-cl1/FAE model

After 20 minutes of β-glucan incubation, no differences in transport were detected (Caco-2-cl1: 7.42 ± 0.39 Log_2_ fluorescence intensity, FAE: 7.46 ± 0.38), however, 1 hour incubation showed a higher passage of β-glucan through the FAE model compared to Caco-2-cl1 monoculture *P* < 0.05 (Fig. 6A).

Confocal microscopy revealed equal amounts of intracellular β-glucan in both models after 20 minutes incubation (Caco-2-cl1: 18.1 ± 0.69 Log_2_ fluorescence intensity, FAE: 18.0 ± 0.57) as well as after 1 hour incubation (Fig. 6B). Translocating β-glucan identified in Caco-2-cl1 and FAE model by confocal microscopy is illustrated in Fig. 6C. All fluorescence intensity values were analyzed on log_2_-scale with the Caco-2-cl1 used as control group. Passage results are based on 7 independent experiments with duplicates, and internalization results on 3 (with 7 Z-stack pictures each).

#### Effects of MβCD and CPZ on Yeast-derived β-glucan Transport

After 20 minutes of pre-incubation with the lipid raft inhibitor MβCD, a lower transport of β-glucan was detected in the FAE model, *P* < 0.05, and a trend towards lower transport also in the Caco-2-cl1 model, *P* = 0.079 (Fig. 6D). However, after 1 hour, only a weak trend remained towards decreased transport of β-glucan in both models (Fig. 6E).

No effect was shown by CPZ, neither after 20 minutes (Caco-2-cl1: nontreated 7.19 ± 0.18 Log_2_ fluorescence intensity, treated 7.17 ± 0.17; FAE: nontreated 7.27 ± 0.16, treated 7.27 ± 0.16) nor after 1 hour incubation (Caco-2-cl1: nontreated 7.30 ± 0.28, treated 7.26 ± 0.25; FAE: nontreated 7.39 ± 0.17, treated 7.45 ± 0.22). Data of fluorescence intensities were analyzed on log_2_-scale and compared to nontreated Caco-2-cl1/FAE models. Results were based on 4 independent experiments with duplicates for each endocytosis inhibitor.

#### Effects of β-glucan on β-hexosaminidase Release

**FIGURE 7.**
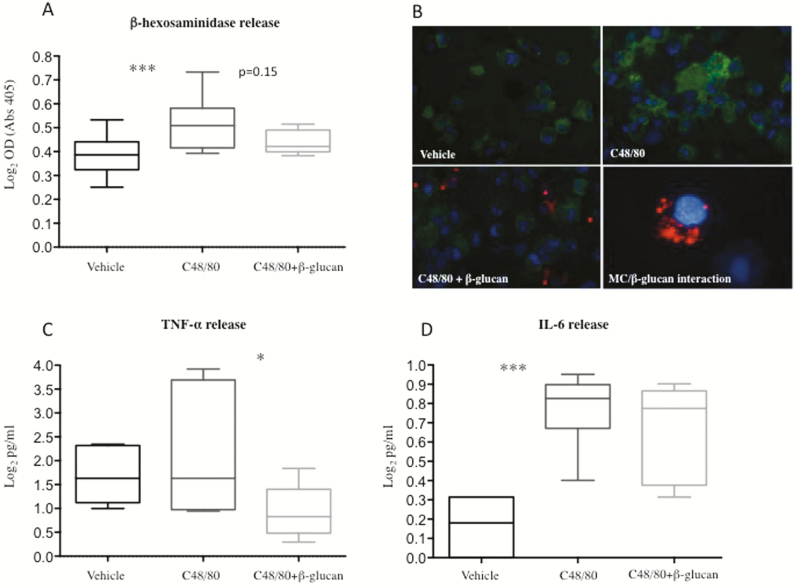
Effects of compound 48/80 (C48/80) and yeast-derived β-glucan on degranulation and cytokine secretion of the human mast cell line HMC-1.1. Degranulation was measured by the release of β-hexosaminidase, TNF-α and IL-6 after 20 minutes of preincubation with β-glucan and 24 hours incubation with C48/80. (A) Stimulation with C48/80 significantly increased β-hexosaminidase release compared to vehicle. Preincubation with β-glucan showed a nonsignificant trend towards decreased degranulation compared to cells stimulated only with C48/80. Results were based on 9 independent experiments with duplicates. (B) Illustration of MC tryptase staining (green) of HMC-1.1 in unstimulated cells (vehicle), cells stimulated with C48/80 and cells incubated with β-glucan (red) prior C48/80-stimulation. The zoomed photograph in the lower right corner illustrates β-glucan in close proximity to a MC, visualized by DAPI nucleus staining (blue). Overview photographs are in 600X magnification and zoomed photograph in 1000X. (C) Stimulation with C48/80 gave no significantly increased levels of TNF-α, however, 20 minutes of preincubation with β-glucan before administration of C48/80 resulted in significantly lower levels of TNF-α compared to C48/80 stimulation only. (D) Stimulation with C48/80 resulted in significantly higher levels of IL-6 compared to vehicle, however, prestimulation with β-glucan had no effect on C48/80-induced degranulation. ***P < 0.001, *P < 0.05. All data were analyzed on log_2_-scale

A significant effect of C48/80 on MC degranulation was confirmed in HMC-1.1 cells by measurement of higher β-hexosaminidase levels after 24 hours of stimulation with C48/80, compared to unstimulated cells, *P* < 0.001 (Fig. 7A). Preincubation with β-glucan prior incubation with C48/80 resulted in a nonsignificant trend, *P* = 0.15, of lower release of β-hexosaminidase compared to cells stimulated with C48/80 only (Fig. 7A). Figure 7B illustrates HMC-1.1 and fluorescence labeled β-glucan.

Stimulation of HMC-1.1 with β-glucan only showed no difference in β-hexosaminidase secretion compared to vehicle. Positive control showed increased β-hexosaminidase release compared to vehicle (1.70 ± 0.09 Log_2_ pg/ml vs. 0.39 ± 0.08), *P* < 0.001. Results were based on 9 independent experiments with duplicates for each condition.

#### Effects of β-glucan on Cytokine Secretion

Stimulation with C48/80 had no significant effect on TNF-α secretion, however, prestimulation with β-glucan 20 minutes before stimulation with C48/80 resulted in lower levels, *P* < 0.05, of secreted TNF-α compared to C48/80 only (Fig. 7C). The IL-6 release was increased after C48/80 stimulation compared to vehicle, *P* < 0.001 (Fig. 7D), though, prestimulation with β-glucan 20 minutes before administration of C48/80 did not result in any significant difference compared to C48/80. Stimulation of HMC-1.1 with only β-glucan gave cytokine levels equal to vehicle. Positive control showed increased TNF-α (6.98 ± 0.42 Log_2_ pg/ml vs. vehicle 1.70 ± 0.61, *P* < 0.01) and IL-6 levels (1.9 ± 0.17 Log_2_ pg/ml vs. vehicle 0.17 ± 0.14, *P* < 0.001). The results for both cytokines were based on 6 independent experiments with duplicates for each condition, respectively.

## DISCUSSION

This study demonstrated for the first time that a yeast-derived β-glucan is able to decrease C48/80-induced hyperpermeability in ileal FAE and VE in patients with Crohn’s ileitis and non-IBD controls. Our findings showed that both paracellular and transcellular permeability increased after C48/80 administration, although in FAE, the increase in transcellular passage was less significant compared to paracellular permeability, and there was only a trend to increased transcellular passage by C48/80 in FAE of CD patients. It is not likely that this is due to the lack of MCs in FAE, since we previously showed that the Peyer’s patches demonstrated MCs at follicle margins, in the adjacent villi, and inside the follicle,^41^ and there was a pronounced effect of C48/80 on paracellullar permeability. It might rather be that MC degranulation is not affecting transcellular passage in FAE to the same extent as paracellular, perhaps due to the different cell composition of FAE and underlying Peyer’s patches.^7,8^ Also, when looking at the C48/80-effects individually (shown in Supplementary data), it was revealed that approximately 60% of the CD patients and 67% of control subjects displayed a pattern similar in FAE to that observed for paracellular permeability.

Our present findings of an increased FITC-dextran passage after C48/80-stimulation could be explained by the release of proinflammatory cytokines such as TNF-α from MC-granules. Several studies have shown TNF-α to weaken the barrier through mechanisms involving tight junction disruption.^17,42^ Studies have shown that anti-TNF-α treatment in CD patients inhibits both the pathological TNF-α and decreases intestinal permeability.^43,44^ In the present study, the effect of the stressor C48/80 on HRP passage was similar in CD and controls in both VE and FAE. One could have expected a more pronounced effect in CD patients due to the higher numbers of MCs found in CD patients,^22^ but MC degranulation might not have affected the transcellular pathways in CD mucosa, but rather the paracellular pathway. This is supported by our findings showing increased baseline paracellular permeability in CD patients, which might refer to the enhanced amounts of degranulated MCs and circulating TNF-α in CD mucosa.

Although oral supplementation of β-glucan could be subject to processing during small bowel transit, which has not been taken into account in this study, it is important to remember that intestinal barrier function is maintained not only by the transepithelial permeability, but also by other factors. For example, the processing of β-glucan by mucosal-adherent microbiota might influence the effects from C48/80 on intestinal permeability. However, most microbial fermentation takes place in the colon whereas the small intestine has a significantly lower amount of bacteria,^45^ suggesting our findings reflect the direct effects of the β-glucan on the epithelium. Addition of β-glucan together with C48/80 in Ussing chambers inhibited the increased FITC-dextran and HRP passage, indicating an effect on both paracellular and transcellular pathways. The effect of β–glucans on C48/80-induced hyperpermeability was similar in CD and controls, which points to resembling mechanisms during inflammation and normal conditions.

To our knowledge, previous studies on β-glucan effects during human inflammation are lacking, however, recently it was shown that the *Saccharomyces cerevisiae* yeast strain and its derivatives prevented colitis induced by adherent invasive *E. coli* in a mouse model mimicking CD.^46^ This interesting finding indicates that β-glucan not only improves barrier function, but might also have a positive effect on inflammation.

Ussing chamber experiments further demonstrate that the inhibitory effect of β-glucan was more pronounced in FAE compared to VE, in CD patients and controls, indicating different mechanisms in uptake of β-glucan in the two epithelial types. The pronounced effect of β–glucan in FAE was confirmed by in vitro experiments showing a significantly enhanced passage of β–glucan through FAE coculture model compared to Caco-2-cl1. The enhanced passage might refer to the presence of M cells in FAE. It has previously been shown^47^ that β-glucan uptake into murine Peyer’s patches was partly M cell-dependent, which is in line with our findings of an increased β-glucan passage in the FAE model compared to Caco-2-cl1, and there also is a more pronounced effect of β-glucan in human FAE compared to VE.

The uptake mechanisms and transport routes for β-glucan are mostly unknown. Studies have shown that uptake and transportation of β-glucan to murine spleen, lymph nodes, and bone marrow involve macrophages,^28^ but uptake of β-glucan by the human epithelium is to our knowledge unidentified. To study mechanism of β-glucan uptake, in vitro experiments were performed using two common endocytosis inhibitors. Lipid rafts are specialized microdomains enriched in cholesterol and glycosphingolipids, and it is known that pathogens like *E. coli* exploits lipid rafts in the plasma membrane to gain entry to the cells.^48^

Preincubation with the lipid raft inhibitor MβCD showed a significant reduction in β-glucan transportation after 20 minutes, but only a trend towards significantly inhibited transport through the FAE model after 1 hour, making it difficult to draw any conclusions of possible long-term transportation mechanisms. Although the results might indicate that β-glucan could be taken up via mechanisms involving lipid rafts in FAE. Another study showed that MβCD could strongly impair the entry of the β-glucan expressing fungi *Candida albicans* into human monocytes by disrupting lipid raft formation in cell membranes.^39^ CPZ is a more specific endocytosis inhibitor compared to MβCD. It inhibits endocytosis by a reversible translocation of clathrin and its adapter proteins, thus, hindering clathrin-mediated vesicle formation.^40^ In the present study, there was no significant effect of CPZ on β-glucan uptake, indicating that β-glucan uptake is not clathrin-dependent.

Using microscopy, we demonstrated that β-glucan could be taken up by the ileal epithelium and pass through the lamina propria. Immunofluorescence revealed β-glucan in close proximity to MCs, macrophages, and DCs in both VE and FAE, and direct colocalization could be observed, especially between β-glucan and macrophages/DCs. Previous studies on β-glucan uptake are lacking, however, macrophages have been shown to be involved in breaking down the orally administered β-glucan to fragments with higher bioactive capabilities.^27^ In addition, De Jesus et al^49^ showed that fluorescent β-glucan particles are taken up in the subepithelial dome of murine Peyer’s patches by a special subset of DCs, DCs^CD11c+^, and that the uptake was partly M cell-dependent, which is in line with our findings of a more pronounced effect of β-glucan in FAE compared to VE. In the present study, we stained for DC-SIGN instead of CD11c, since DCs^CD11c+^ are more frequent in the human colon.^50^ We found β-glucan in close proximity to DCs, MCs, and macrophages. Direct colocalization of β-glucan was generally seen with DCs and macrophages, which strengthens the importance of these cells in β-glucan translocation. Both MCs and macrophages were identified close to the epithelial cells where β-glucan was present, indicating a close interaction between β-glucan and cells at an early stage. Our results further showed significantly higher numbers of MCs, macrophages, and DCs in the VE of CD patients compared to controls, highlighting the presences of an inflammatory state in CD. A significantly higher percentage of both macrophages and DCs were found colocalizing/in close proximity to β-glucan in VE of CD compared to controls. This together indicates not only a higher uptake of β-glucan in CD, but also that β-glucan interacts with immune cells to a higher extent in CD compared to controls. Whether these findings are a contributing factor to the attenuated MC-induced hyperpermeability after β-glucan stimulation is not known. However, our findings do broaden the knowledge about the internalization capability of both macrophages and DCs for fungi-associated components in CD patients. Previous studies in this field are to our knowledge mostly lacking, however, in line with our results, Vazeille et al showed that macrophages from CD patients have a higher internalization rate of *E. coli* compared to healthy controls.^51^ The capability of DCs to interact with β-glucan has been well described,^47,49^ but their internalization rate in inflammatory conditions such as CD is to our knowledge less known. Our results could prove useful and important for future studies investigating the relation of the mycobiota and pathogenesis of CD.^52^

Our ex vivo results may suggest that β-glucan end up juxtaposed to MCs and block their degranulation, resulting in the observed attenuation of C48/80-induced hyperpermeability.

We initially hypothesized that the attenuation of the C48/80-induced hyperpermeability, with the use of yeast-derived β-glucan, was achieved by inhibition of MC-degranulation, thereby, having prevented the release of eg, TNF-α. However, in vitro experiments with HMC-1.1 cells could not fully support this hypothesis, as preincubation with β-glucan before stimulation with C48/80 only had a nonsignificantly lowering effect on MC degranulation. Moreover, C48/80 did not significantly increase the TNF-α levels compared to vehicle, however, prestimulation with β-glucan showed a significant decrease in comparison to C48/80 only. This could imply that yeast-derived β-glucan has some stabilizing effect on constitutive TNF-α release, as HMC-1 are known to be continuously active.^53,54^ Measurement of IL-6 secretion from HMC-1.1 showed a significant increase after C48/80 stimulation but preincubation with β-glucan failed to reduce IL-6 secretion. These inconclusive in vitro data could suggest that β-glucan exerts its barrier protective effects through unknown MC-independent mechanisms, but it is also important to remember that the in vitro setting does not completely mirror the in vivo situation. For example, it has been shown that β-glucan undergoes processing by macrophages.^27^ Our results show colocalization of β-glucan to both macrophages and DCs, possibly resulting in the release of fragmented particles in processed forms that have other properties compared to their original form and, thereby, exerts more potent effects on MCs. However, this was not investigated in this study.

Interestingly, one human clinical study^55^ showed that 4 weeks of daily oral supplementation with 250 mg Baker’s yeast-derived β-glucan decreased total ragweed allergy symptoms and severity. These findings did not correlate with changes in IgE levels and ,therefore, could suggest an attenuation of MC-degranulation since allergic reactions are highly dependent on MC- degranulation.^56^

In conclusion, we provide evidence that yeast-derived β-glucan has a beneficial effect on ileal barrier function by inhibiting stress effects on the epithelium. β-glucan was able to attenuate paracellular and transcellular hyperpermeability caused by MC-degranulation*. In vitro* studies showed that β-glucan passed through the FAE at a higher rate compared to VE, and the uptake seemed to involve lipid raft formation. Our results provide important and novel knowledge, and highlight the possible application of yeast-derived β-glucan in health disorders and diseases characterized by intestinal barrier dysfunction, such as CD.

### Statement of authorship

Åsa V. Keita, PhD and Ida Schoultz, PhD designed the research; John-Peter Ganda Mall, MSc, Maite Casado-Bedmar, MSc, Martin E. Winberg, PhD, and Dr. Keita conducted the research; Drs. Schoultz, Keita, Robert J. Brummer, MD, PhD, and Mr. Ganda Mall and Ms Casado-Bedmar analyzed data; Drs. Schoultz and Keita and Mr. Ganda Mall and Ms. Casado-Bedmar wrote the paper; Dr. Keita had the primary responsibility of the final content. All authors read and approved the final manuscript.

## SUPPLEMENTARY DATA

Supplementary data is available at *Inflammatory Bowel Diseases* online.

Supplementary Figure 1

Supplementary Figure 2

Supplementary Table 1
