# Supplementary figures and images for: A β-Glucan-Based Dietary Fiber Reduces Mast Cell-Induced Hyperpermeability in Ileum From Patients With Crohn’s Disease and Control Subjects
Source: Inflamm Bowel Dis. 2017 Dec 19;24(1):166–78. doi: 10.1093/ibd/izx002 (PMC6166688; doi:10.1093/ibd/izx002)

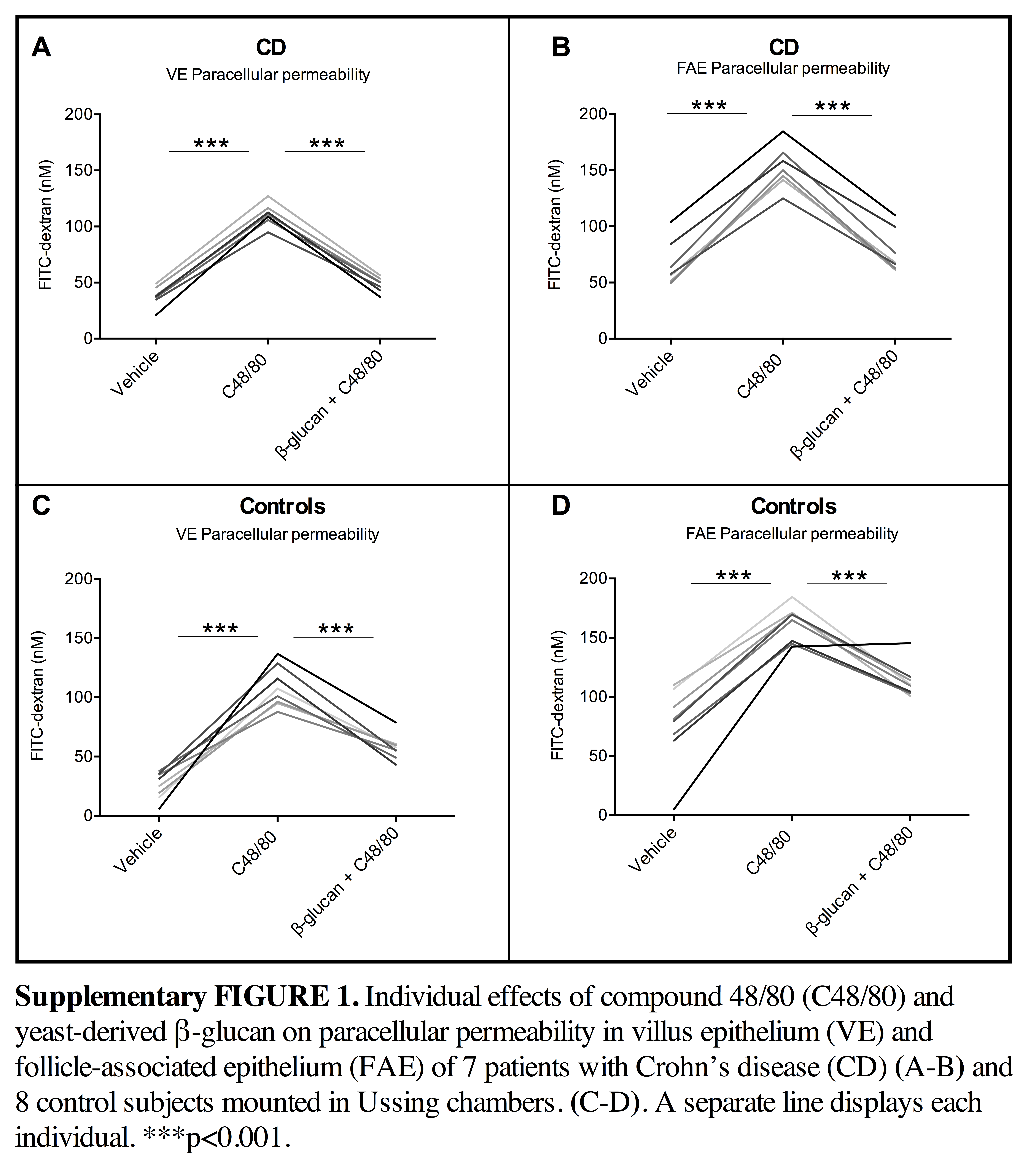

Supplement: Supplementary Figure 1 [file izx002_suppl_supplementary_figure_1.png]

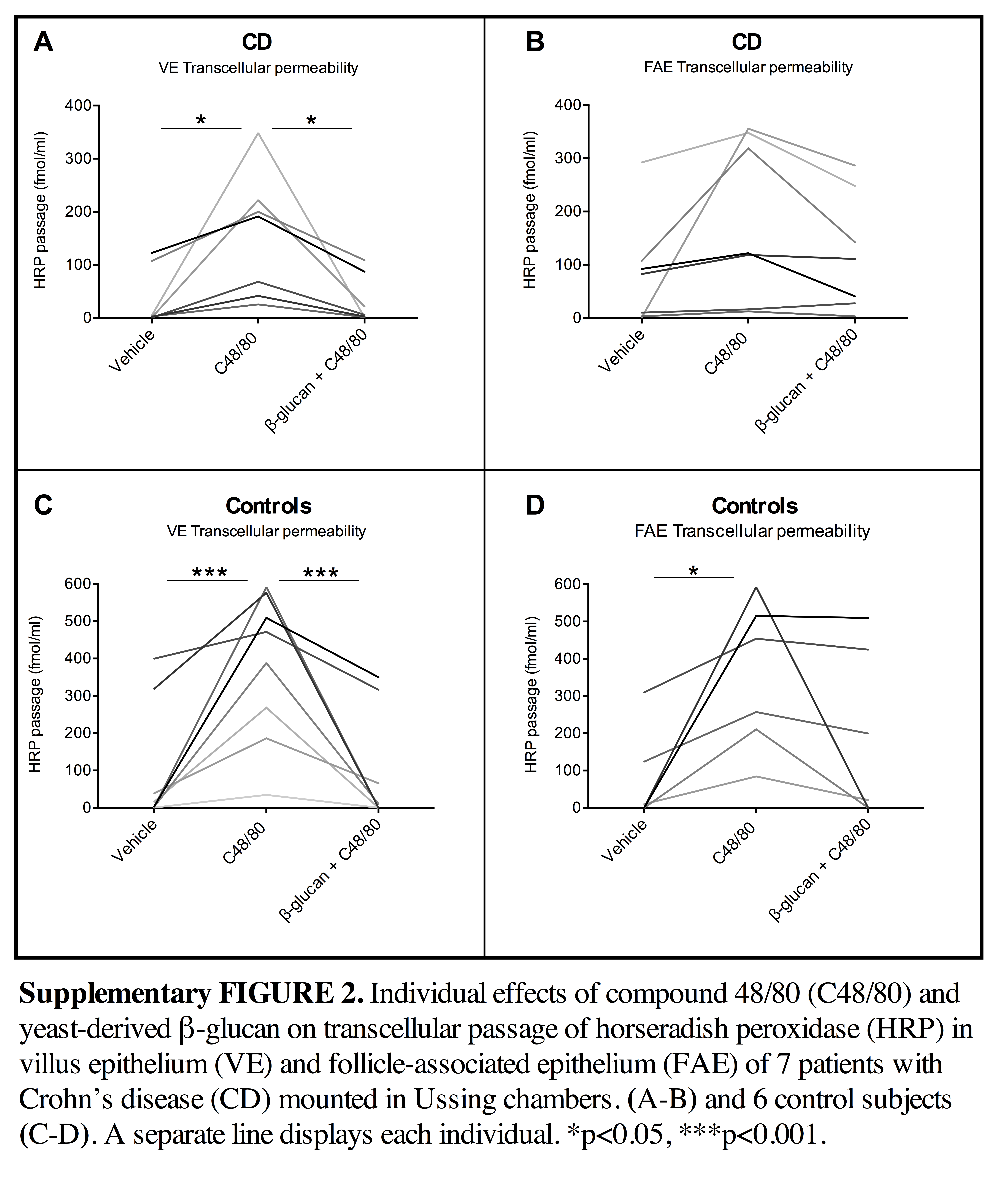

Supplement: Supplementary Figure 2 [file izx002_suppl_supplementary_figure_2.png]
