# Supplementary material for: A β-Glucan-Based Dietary Fiber Reduces Mast Cell-Induced Hyperpermeability in Ileum From Patients With Crohn’s Disease and Control Subjects
Source: Inflamm Bowel Dis. 2017 Dec 19;24(1):166–78. doi: 10.1093/ibd/izx002 (PMC6166688; doi:10.1093/ibd/izx002)
Supplement: Supplementary Table 1 [file izx002_suppl_supplementary_table_1.docx]

**Supplementary Table 1.** Short circuit current (Isc) and transepithelial resistance (TER) in human ileum.

|  | **Isc** (μA/cm^2^) | | **TER** (Ohm/cm^2^) | |
| --- | --- | --- | --- | --- |
| **Non-IBD controls** | **VE** | **FAE** | **VE** | **FAE** |
| Vehicle | 13.3 ± 3.1 | 9.2 ± 2.3 | 85.3 ± 7.2 | 89.9 ± 6.2 |
| C48/80 | 15.6 ± 3.2 | 12.1 ± 3.0 | 78.8 ± 8.2 | 81.3 ± 4.9 |
| β-glucan + C48/80 | 13.2 ± 2.1 | 9.5 ± 1.7 | 83.0 ± 6.5 | 87.6 ± 5.2 |
| **Crohn’s disease** | **VE** | **FAE** | **VE** | **FAE** |
| Vehicle | 14.2 ± 5.1 | 11.7 ± 2.2 | 88.9 ± 2.4 | 89.3 ± 4.2 |
| C48/80 | 17.1 ± 4.2 | 14.2 ± 2.4 | 81.8 ± 2.2 | 81.5 ± 2.5 |
| β-glucan + C48/80 | 13.4 ± 2.8 | 10.6 ± 3.3 | 87.9 ± 2.7 | 88.1 ± 2.3 |

Segments of villus epithelium (VE) and follicle-associated epithelium (FAE) from 8 non-inflammatory bowel disease (IBD) controls and 7 patients with Crohn’s disease were mounted in Ussing chambers and exposed to vehicle, mast cell degranulator compound 48/80 (C48/80) or C48/80 + yeast-derived β-glucan. Comparisons were done with ANOVA. There were no significant effects on Isc and TER by C48/80 and/or β-glucan.
